# The mechanisms and diagnostic potential of lncRNAs, miRNAs, and their related signaling pathways in cervical cancer

**DOI:** 10.3389/fcell.2023.1170059

**Published:** 2023-05-04

**Authors:** Yan Xu, Yu Sun, Xiaobin Song, Jian Ren

**Affiliations:** Shandong University of Traditional Chinese Medicine, Jinan, China

**Keywords:** lncRNAs, miRNAs, cervical cancer, signaling pathways, lncRNA–miRNA

## Abstract

Cervical cancer (CC), the fourth most prevalent type of cancer among women worldwide, is associated with high rates of morbidity and mortality. Due to the long period of latency in CC, most patients are already in the middle to late stages when initially diagnosed, which greatly reduces the clinical cure rate and quality of survival, thus resulting in poor outcomes. In recent years, with continuous exploration in the fields of bioinformatics and molecules, it has been found that ncRNAs, including miRNAs and lncRNAs, without the ability to translate proteins are capable of activating or inhibiting certain signaling pathways by targeting and modulating the level of expression of proteins involved in these signaling pathways. ncRNAs play important roles in assisting with diagnosis, drug administration, and prediction of prognosis during CC progression. As an entry point, the mechanisms of interaction between miRNAs, lncRNAs, and signaling pathways have long been a focus in basic research relating to CC, and numerous experimental studies have confirmed the close relationship of miRNAs, lncRNAs, and signaling pathways with CC development. Against this background, we summarize the latest advances in the involvement of lncRNA- and miRNA-related signaling pathways in the development of CC to provide guidance for CC treatment.

## 1 Introduction

Cervical cancer (CC), as the fourth most common type of cancer worldwide in women, represents a serious threat to the health of the female reproductive system. In particular, the incidence and mortality of CC are significantly higher in developing countries than in developed countries (Bray et al., 2018; Cohen et al., 2019). Squamous cell carcinoma is the most prevalent among all histological types, comprising approximately 70% of cases, followed by adenocarcinoma at 20% (Small et al., 2017). Human papillomavirus (HPV) infection is the main cause of the development of CC. It has been noted that more than 70% of CC cases worldwide are caused by HPV types 16 and 18 (Cubie, 2013). HPV is a small, double-stranded, closed-loop DNA virus whose genome contains three functional regions (a protein-coding region, a late protein-coding region, and a non-coding region), of which E6 and E7, which belong to the protein-coding region, are the predominant oncogenic proteins (Oyouni, 2023). E6- and E7-induced alterations in transcriptional regulation lead to genomic instability, thereby distinguishing the cellular transformation process from productive viral infection, a process that represents an important subsequent step in malignancy (Snijders et al., 2006). Constitutive expression of E6/E7 immortalizes primary epithelial cells and promotes tumor formation *in vivo* (Yang and Al-Hendy, 2022). E6 also mediates binding to p53 via E6-related proteins, thereby promoting ubiquitinated degradation of p53 and malignant cellular progression. E7 binds to cell cycle protein-dependent kinase inhibitors, leading to loss of cell cycle control (Oyouni, 2023). Although HPV screening and vaccination have greatly reduced the prevalence of CC (Zur Hausen, 2002; Canfell, 2019; Zhang and Batur, 2019), CC survival rates remain unsatisfactory at present, because most patients with cervical cancer are diagnosed at an advanced stage or a stage at which distant lymph node metastases are present (Dong et al., 2018). Therefore, it is particularly important to identify early diagnostic and prognostic biomarkers of CC. With the development of genome sequencing technologies, including high-throughput microarrays and single-cell sequencing, the role of non-coding RNAs (ncRNAs) in physiological processes and in disease progression has been in focus (Koffler-Brill et al., 2023). Recent studies have confirmed that aberrant expression of long non-coding RNAs (lncRNAs) is an essential factor in the development of CC, thus endowing lncRNAs with the potential to be used as the latest diagnostic biomarker for CC (Dastmalchi et al., 2022). In fact, the role of lncRNAs in CC has already been fruitfully investigated. Gibb et al. analyzed 16 long serial analyses of gene expression (L-SAGE) libraries composed of cervical specimens and also identified the expression profile of 1,056 lncRNAs in the human cervix, thus presenting the first lncRNA expression profile of the cervix (Gibb et al., 2012). More importantly, it was further determined that changes in lncRNA expression do occur in cervical intraepithelial lesions, indicating aberrant expression in early tumors (Gibb et al., 2012). lncRNAs and microRNAs (miRNAs) belong to the class of ncRNAs, which can interfere with tumorigenesis, tumor invasion, and metastasis by regulating epigenetic, transcriptional, and post-transcriptional processes, cell proliferation and differentiation, apoptosis, and autophagy (Bravo-Vázquez et al., 2023). Currently, the interplay of lncRNA and miRNA with tumor-related signaling pathways is a hotspot in the diagnosis and treatment of tumors of the reproductive system (Lai et al., 2022; Ma et al., 2022). Here, we review the mechanisms of lncRNA- and miRNA-related signaling pathways in CC.

## 2 Mechanisms relating to lncRNAs in CC

### 2.1 Biological functions of lncRNAs

**FIGURE 1:**
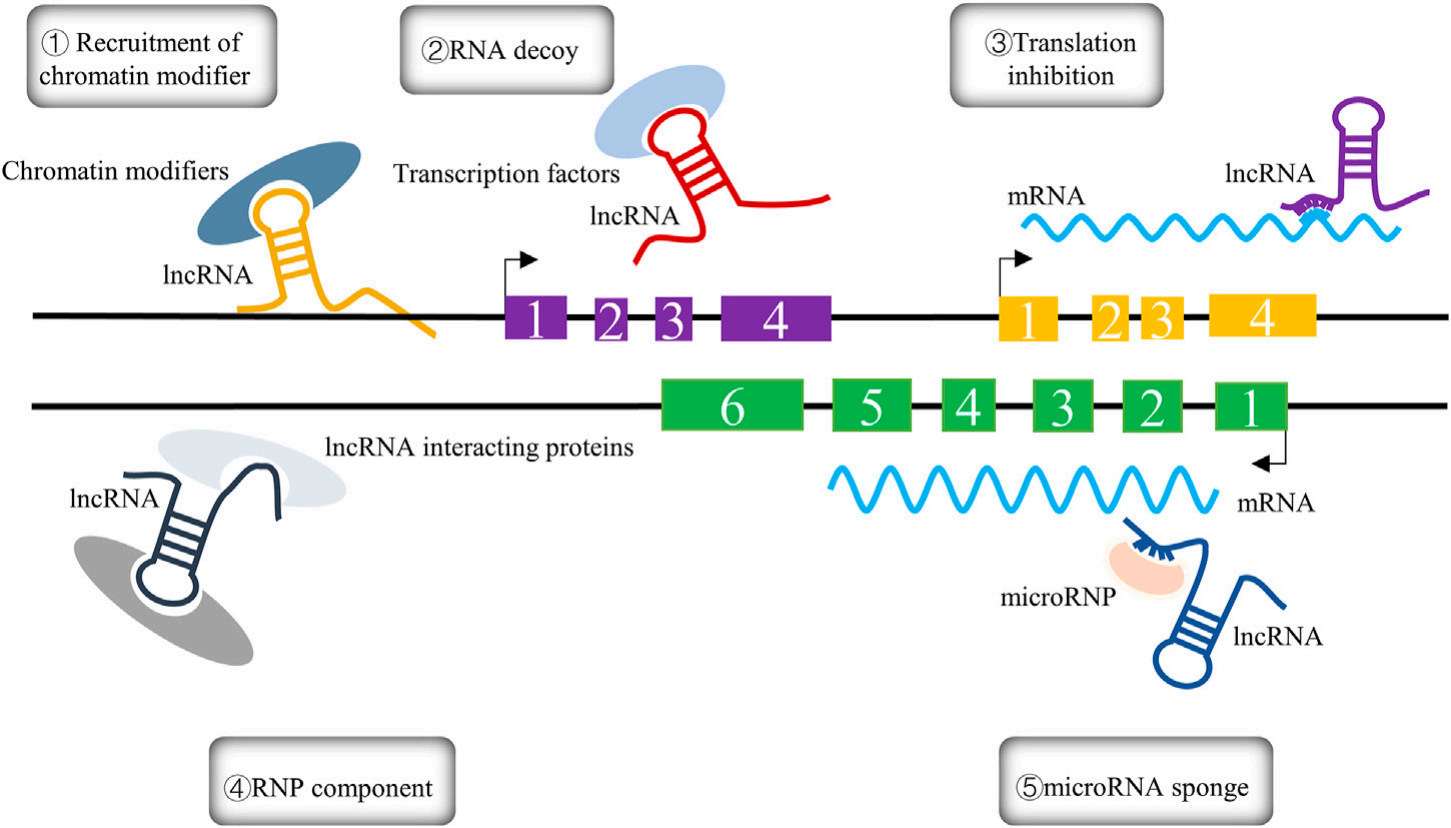
The five different mechanisms of lncRNAs are summarized in the figure and can be expressed as follows: ① Recruitment of chromatin modifiers; ② RNA decoy; ③ translation inhibition; ④ RNP component; ⑤ microRNA sponge.

lncRNAs, as RNA molecules with transcripts longer than 200 nucleotides, cannot be translated into functional proteins; they are characterized by low expression, high stability, and poor interspecies conservation, and were initially considered to be too “noisy” for genomic transcription due to their inability to encode protein information molecules (Mattick and Rinn, 2015; Chi et al., 2019; Liu et al., 2021a; Zangouei et al., 2023). With the advent of deep genomics studies, lncRNAs were found to be involved in diverse biological processes, such as cell differentiation and growth, and to play a vital role in multiple reproductive diseases (Elsayed et al., 2020). The biological functions of lncRNAs are largely determined by their location in cells. Specifically, lncRNAs located in the nucleus can regulate epigenetic gene expression by recruiting chromatin remodeling and modification complexes to target promoters to promote or repress their transcription in cis or trans regulation (Yap et al., 2010; Holdt et al., 2013); they can act as decoys for specific chromatin-modifying factors to limit the binding ability of transcription factors to DNA-binding sites (Jain et al., 2016); and they can also serve as transcriptional regulators through binding DNA or DNA-binding domains (Kino et al., 2010). In contrast, lncRNAs located in the cytoplasm are involved in post-transcriptional regulation through their influence on the stability of mRNAs (Kumar et al., 2014). lncRNAs can also act as translation factors participating in the regulation of translation in the cytoplasm; additionally, a novel androgen receptor (AR) translation regulator, lncRNA LBCS, can directly interact with hnRNPK to inhibit AR translation by forming a complex with hnRNPK and AR mRNA (Gu et al., 2019). In addition to their direct impact on gene regulation, lncRNAs can serve as natural miRNA “sponges” that release miRNAs from their target genes (Kopp and Mendell, 2018). The multiple roles of lncRNAs are depicted in Figure 1.

Currently, modern medical research based on the mechanism underlying the involvement of lncRNA in CC development mainly focuses on several aspects.

Regulation of autophagic flux. Autophagy is a key biological phenomenon to that aids in the maintenance of cellular homeostasis, with the help of lysosomal degradation, which involves many key factors, including lncRNAs (Jiang and Mizushima, 2014; Bonam et al., 2020). Microtubule-associated protein 1A/1B light chain 3 (MAP1LC3), which is central to the autophagic process, functions as a core that is primarily involved in the formation of autophagic vesicles and is sheared at its carboxyl terminus by autophagy-associated genes (ATG)4 immediately after synthesis to form LC3-I (Maruyama and Noda, 2017; Bonam et al., 2020). It is then activated by APG7L/ATG7, transferred to ATG3, and coupled to phosphatidylethanolamine (PE) to form the membrane-bound form LC3-II (Maruyama and Noda, 2017; Bonam et al., 2020). LC3-II has several functions during autophagy, such as elongation, barrier membrane sealing, and cargo recognition (Maruyama and Noda, 2017). Therefore, LC3-I and LC3-II in autophagosomes have become molecular markers for the occurrence of autophagy: the amount of LC3-II is proportional to the degree of autophagic flux, while the LC3-II/LC3-I ratio reflects the level of autophagy (Bonam et al., 2020). For example, lncRNA-IGFBP4, as an oncogene and a tumor suppressor in lung, prostate, breast, and other cancers, has been found to participate in autophagic response by interacting with c-Myc and to indirectly inhibit autophagy in HeLa cells of CC origin based on the observation of LC3 puncta formed in GFP-LC3 in engineered HeLa cell lines (Zhang et al., 2022a).

Regulation of the cell cycle. He et al. (2021) found that lncRNA DLEU2 can inhibit the Notch signaling pathway by suppressing p53 expression and can ultimately promote cell proliferation in CC cells, suggesting that lncRNA-DLEU2 is involved in CC development through facilitation of the cell cycle.

Regulation of epithelial–mesenchymal transition (EMT) and immune evasion. Liao et al. (2022) demonstrated that an lncRNA known as lymph node metastasis-associated suppressor (LNMAS) suppresses TWIST1-mediated EMT and STC1-dependent immune evasion by weakening the HMGB1–BRG1 interaction, thereby suppressing CC cell growth; this provides an indication of potential therapeutic targets for CC.

Regulation of angiogenesis. Lei and Mou (2020) delivered taurine upregulated gene 1 (TUG1) as an exosome from two CC cell lines (HeLa and CaSki) into human umbilical vein endothelial cells (HUVECs) and found that TUG1 enhanced the proliferation of vascular and endothelial cells by stimulating the ecto-expression of angiogenesis-related genes, including vascular endothelial growth factor (VEGF-A), matrix metallopeptidase 9 (MMP-9), transforming growth factor-β (TGF-β), interleukin (IL-8), and basic fibroblast growth factor (bFGF); this may serve as an early biomarker for CC.

**FIGURE 2:**
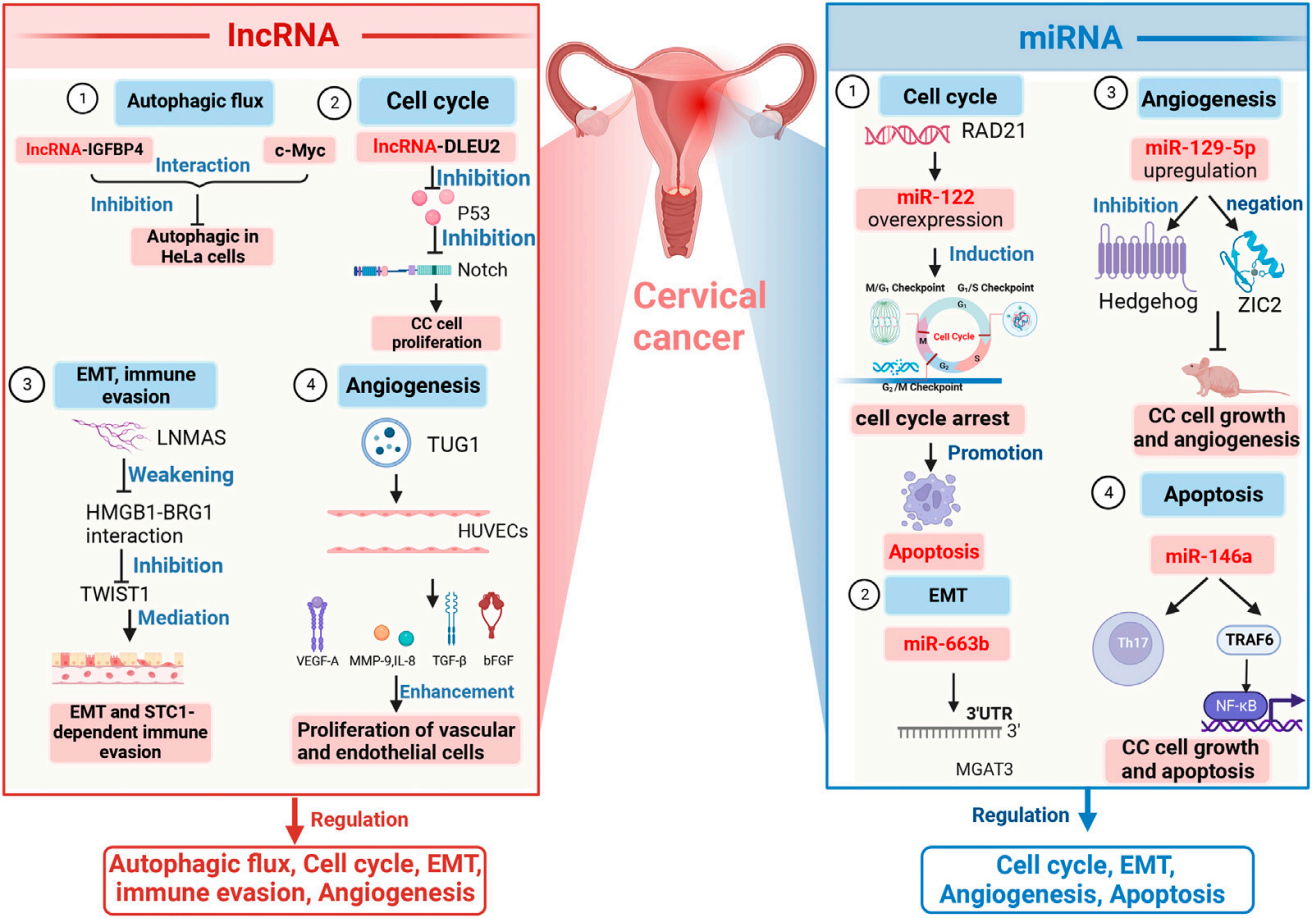
Mechanisms of lncRNAs and miRNAs in CC development. lncRNAs and miRNAs jointly regulate the cell cycle, EMT, and angiogenesis. Regulation of autophagy is the domain of lncRNAs; regulation of apoptosis is one of the functions of miRNAs. Several specific lncRNAs and miRNAs have been shown to be involved in different mechanisms underlying these functions, such as DLEU2, TUG1, miR-122, miR-129–5p, and miR-146. Note: Notch, neurogenic locus notch homolog protein; EMT, epithelial–mesenchymal transition; LNMAS, lymph node metastasis-associated suppressor; STC1, stanniocalcin 1; TWIST1, twist family bHLH transcription factor 1; HUVECs, human umbilical vein endothelial cells; VEGF-A, vascular endothelial growth factor; MMP-9, matrix metallopeptidase 9; TGF-β, transforming growth factor-β; IL-8, interleukin-8; 3′-UTR, 3′-untranslated regions; MGAT3, mannoside acetylglucosaminyltransferase 3; ZIC2, zinc finger of the cerebellum protein 2; Th17, T helper cell 17; NF‐κB, nuclear factor kappa-light-chain-enhancer of activated B cells; TRAF6, tumor necrosis factor receptor-associated factor-6; lncRNA-IGFBP4, lncRNA insulin-like growth factor-binding protein 4; lncRNA-DLEU2, lncRNA deleted in lymphocytic leukemia 2; HMGB1-BRG1, high-mobility group protein 1-brahma-related genes 1; TUG1, taurine upregulated gene 1.

The mechanisms underlying the role of lncRNA in CC development are depicted in Figure 2.

### 2.2 The role of lncRNAs in CC diagnosis and prognosis

Early and effective diagnosis is very important for the treatment prospects of all cancers. It has been demonstrated that lncRNAs are vital in controlling nuclear structure and transcription in the nucleus and in modulating stability and translation, which are regarded as potential diagnostic biomarkers for cancer metastasis (Chi et al., 2019; Yao et al., 2019). CC is one of the most common gynecological cancers and is mostly diagnosed at late stages due to deficiencies in screening strategies for this form of cancer. Therefore, it is essential to develop a comprehensive understanding of the potential molecular mechanisms in CC in order to explore potential therapeutic targets and improve the prognosis of CC patients (Thankachan et al., 2021). For example, MIR210HG and ABHD11-AS1 are two lncRNAs found to be upregulated in CC. Wang et al. (2020) identified MIR210HG as one of the most upregulated lncRNAs in CC by analyzing GEO array data and found that the level of expression of MIR210HG is associated with the clinical characteristics and prognosis of advanced CC. In addition, a loss-of-function assay revealed that downregulation of MIR210HG suppresses tumor cell proliferation, invasion, and metastasis in CC (Wang et al., 2020). Hu et al. found that the hypoxia-induced lncRNA MIR210HG was overexpressed in CSCC tissues and regulated by HPV type 16 E6 and E7 via hypoxia-inducible factor 1α (HIF-1α); through further functional assays, they also showed that MIR210HG promotes CSCC cell proliferation, migration, and invasion *in vitro* under both normoxic and hypoxic conditions (Hu et al., 2022a). ABHD11-AS1, an important promoter in human malignancies, is located at 7q11.23 and is known as the long intergenic non-coding RNA 35 (LINC00035) and Williams–Beuren syndrome chromosome region 26 (WBSCR26) RNA gene. A number of studies in recent years have shown that ABHD11-AS1 is a potential biomarker for early diagnosis of CC (Golla et al., 2022). Zhu et al. (2022) observed increased lncRNA ABHD11-AS1 and decreased miR-1254 in the serum of CC patients compared with healthy controls, suggesting that dysregulation of ABHD11-AS1 and miR-1254 is involved in CC progression. Further investigation of the corresponding mechanisms revealed a regulatory correlation between lncRNA ABHD11-AS1 and miR-1254. Silencing of lncRNA ABHD11-AS1 was found to promote miR-1254 expression, while overexpression of lncRNA ABHD11-AS1 was found to inhibit miR-1254 expression. Therefore, the authors speculated that lncRNA ABHD11-AS1 affects CC cell viability through regulation of miR-1254, suggesting that lncRNA ABHD11-AS1 may serve as a new therapeutic target for CC treatment. Yang et al. (2022a) evaluated the expression of LAMTOR5-AS1 in cervical cancer tissues and cells by polymerase chain reaction (PCR). The effect of LAMTOR5-AS1 on proliferation, migration, and invasion of CC cells was also verified by cell counting kit-8 (CCK-8) and transwell assays. Moreover, luciferase reporter gene assay indicated that LAMTOR5-AS1 inhibits CC proliferation via a sponge effect on miR-210–3p, and a negative correlation was observed between LAMTOR5-AS1 and miR-210–3p (Yang et al., 2022a), supporting LAMTOR5-AS1 as a biomarker for CC. Hou et al. (2020) observed that MAGI2-AS3 exerted a sponge effect on miR-233 in order to upregulate EPB41L3 after transfecting MAGI2-AS3, thereby promoting the invasion and migration of CSCC cells. Finally, in a recent study, Chai et al. (2022) found that MAGI2-AS3 expression is negatively linked with miR-15b in cervical cancer tissues and that miR-15b partially reverses the promoting effect of MAGI2-AS3 on HeLa cell viability and invasion. Luciferase reporter assay revealed that miR-15b directly binds to the 3′ UTR of CCNE1 and that upregulation of miR-15b inhibits CCNE1 expression in HeLa cells. Meanwhile, overexpression and downregulation of MAGI2-AS3 could enhance and suppress CCNE1 expression, respectively. Therefore, it could be inferred that MAGI2-AS3 suppresses CC growth and invasion through the miR-15b/CCNE1 pathway, and MA-GI2-AS3 can be considered an effective target for CC diagnosis.

The above-described studies suggest that lncRNAs can be used as important diagnostic biomarkers for CC, but further evidence is still required to support this view. In the following sections, we divide CC-related lncRNAs into two categories (pro-tumor lncRNAs and anti-tumor lncRNAs); we summarize their biological functions and mechanisms in Table 1 and Table 2.

**TABLE 1:** Ectopic expression of oncogenic lncRNAs in CC.

lncRNA	Expression	Types of CC tissues or cell lines studied	Biological function	Mechanism	Reference
HOXA-AS3	Up	132 patients (low-expression group tissue and high-expression group tissue)	Promotes proliferation, metastasis, and invasion of CC	Negatively regulates CC development by sponging miR-29a-3p	Xu et al. (2022a)
LINC00511	Up	53 patients (CC tissue and adjacent normal tissue)	Promotes proliferation, metastasis, and invasion of CC cells	Targets miR-497–5p and upregulates MAPK1 expression	Lu et al. (2022)
LINC01287	Up	80 CC tissue samples and adjacent normal tissue samples	Promotes proliferation of CC cells, colony formation, migration, and apoptosis	Positively regulates SERP1 expression by sponging miR-513a-5p	Hu et al. (2022b)
HIF1A-AS2	Up	CC tissue and non-cancerous cervical tissue; cells (CaSki, SiHa, HeLa, and C33A)	Promotes CC cell proliferation, migration, and invasion; inhibits CC cell apoptosis	Mediated by HPV16 E6; regulation of cell apoptosis via the P53/caspase 9/caspase 3 axis	Guan et al. (2022a)
LINC00649	Up	127 patients (low-expression group tissue and high-expression group tissue)	Promotes CC cell proliferation abilities, migration capacity, and invasive power	Aggravates CC progression by targeting miR-216a-3p	Si et al. (2022)
SCIRT	Up	34 tumor tissue samples and 34 tumor-adjacent tissue samples	Promotes the proliferative, migratory, and invasive properties of CC cells	Upregulates MMP-2/MMP-9	Guan et al. (2022b)
UCA1a	Up	Human CC cell line HeLa	Promotes CC proliferation	Increases PKM2 protein level by binding to PKM2 protein and enhancing its stability	Yu et al. (2022)
LOXL1-AS1	Up	50 paired CC tissue and non-cancerous tissue samples; cells (HeLa, CaSki, C33A, SiHa, and Ect1/E6E7)	Promotes CC cell proliferation, migration, invasion, and angiogenesis	Sponges miR-526b-5p and regulates LYPLA1	Zhang et al. (2022b)
LINC00707	Up	Cells (H8, SiHa, HeLa, CaSki, and C-33A)	Promotes proliferation of CC cells while inhibiting apoptosis	Sponges miR-374c-5p and upregulates SDC4 expression	(Fang, Guo, Zheng, Li)
OIP5-AS1	Up	50 patients (CC tissue and adjacent normal tissue); cells (End1/E6E7, HeLa, CaSki, SiHa, and ME-180)	Promotes CC cell migration, invasion, and EMT	Suppresses miR-147a expression and activates the IGF1R pathway	Zhang et al. (2022c)
LOC100130075	Up	Cells (SiHa, HeLa, CaSki, C-33A, and H8)	Promotes CC progression	Positively correlates with MDM2 and binds to E2F1 for activation of MDM2	Xu et al. (2022b)
FLVCR1-AS1	Up	Cells (C-4-I, C-33A, SiHa, HeLa, and Ect1/E6E7)	Promotes proliferation, migration, and invasion of CC cells; inhibits apoptosis.	Sponges miR-381–3p and targets MAGT1	Zhang et al. (2022d)
		40 patients (CC tumor tissue, adjacent normal tissue, and serum samples); cells (HUCEC, HeLa, CaSki, C-33A, and AV3)	Promotes proliferation and migration, invasion, and EMT of CC cells; inhibits apoptosis	Competitive binding to miR-23a-5p and promotion of expression of SLC7A11	Zhou et al. (2022)

**TABLE 2:** Ectopic expression of anti-tumor lncRNAs in CC.

lncRNA	Expression	Types of CC tissues or cell lines studied	Biological function	Mechanism	Reference
LAMTOR5-AS1	Down	120 patients (CC tumor tissue and adjacent normal tissue); cells (HeLa, HCE1, CaSki, SiHa, and H8)	Suppresses proliferation, migration, and invasion of CC cells	Negatively regulates expression of miR-210–3p	Yang et al. (2022a)
PTENP1	Down	88 CC tissue samples and matched non-tumor tissue samples; cells (C33A, HeLa, ME-180 and SiHa, and NC104)	Suppresses CC cell growth, motility, and EMT	Acts as a decoy for miR-27a-3p to upregulate EGR1 expression	Wu et al. (2022)
FAM13A-AS1	Down	30 patients (CC tumor tissue and adjacent normal tissue); cells (HeLa, SiHa, and HUCEC)	Suppresses CC cell proliferation, apoptosis, invasion, and migration	Negatively regulates expression of miRNA-205–3p and promotes expression of DDI2	Qiu et al. (2022)
PGM5-AS1	Down	29 patients (CC tissue and adjacent normal tissue); cells (HeLa, CaSki, and HaCaT)	Suppresses CC cell proliferation, migration, and invasion	Sponges miR-4284 to upregulate DCN expression	Wang et al. (2022a)

## 3 MiRNA interference with CC development

### 3.1 Mechanisms for miRNAs

miRNAs are a group of small, single-stranded ncRNAs with 19–23 nucleotides (Li et al., 2018). Most miRNAs bind imperfectly complementary to the 3′ untranslated region (3′ UTR) of their target mRNA, resulting in translational repression or degradation of the mRNA (Smolarz et al., 2022; Kargutkar et al., 2023). The mechanism by which miRNA takes effect is through inhibition of translation assembly: miRNA competes with eIF4E at the m7G cap site of the mRNA, while promoting deadenylation, decapping, and degradation of the mRNA through recruitment of the PAN2-PAN3 complex, the CCR-NOT complex, and exoribonuclease 1 (XRN1) (Kargutkar et al., 2023).

The mechanism of cancer development has still not been fully deciphered, but the imbalance of intracellular homeostasis triggered by epigenetic changes is one of the currently accepted mechanisms (Ge et al., 2022). Epigenetic changes allow tumor cells to spread and subsequently to develop distant metastasis (Su et al., 2022). Epigenetic factors and miRNAs, as regulators of gene expression, interact with each other to form an epigenetic miRNA regulatory circuit in order to maintain normal physiological functions of the body; once this regulatory circuit is disrupted, the risk of cancer is elevated (Sato et al., 2011). Specifically, a particular set of miRNAs (defined as epi-miRNAs) can indirectly affect the expression of tumor suppressor genes by influencing epigenetic mechanism effectors such as DNA methyltransferases, histone deacetylases, and polyoma suppressor complex genes, which are also controlled by epigenetic factors (Fabbri and Calin, 2010). Further information on the mechanism underlying the occurrence of CC based on miRNAs has recently been obtained (Sui et al., 2022). According to current findings, the mechanisms of miRNAs in CC development include the following aspects.

Involvement in cell cycle regulation. Yang et al. (2022b) conducted a bioinformatics analysis to identify the target gene of miR-122 (RAD21) and found that overexpression of miR-122 induces cell cycle arrest and promotes apoptosis through targeting of RAD21, thus participating in the pathological development of CC.

Regulation of EMT. You et al. (2021) revealed that miR-663b can directly target the 3′UTR of monoacylglycerol acyltransferase 3 (MGAT3) and can participate in the EMT regulatory process.

Regulation of angiogenesis. Wang et al. (2018) observed that upregulation of miR-129–5p inhibits CC cell growth and angiogenesis in naked mice through suppression of the Hedgehog signaling pathway and negative targeting of ZIC2.

Regulation of apoptosis. Li et al. (2019a) observed that miR-146a exerts an effect on regulation of Th17 cell differentiation, and further studies have revealed that miR-146a enables its target gene TRAF6 to regulate CC cell growth and apoptosis through the NF-kB signaling pathway.

The mechanisms of miRNAs in relation to CC development are illustrated in Figure 2.

### 3.2 The role of miRNAs in CC diagnosis and prognosis

Given the challenges of CC in terms of its early concealment and the lack of sufficient screening, the development of methods enabling accurate diagnosis of CC at an early stage is valuable. A plethora of studies involving miRNAs have focused on exploring biomarkers for the diagnosis and prognosis of CC. Regarding miRNAs for diagnostic and prognostic purposes, it is crucial to clarify their specific effects on tumors and the ways in which dysregulated miRNAs affect tumor progression (Bañuelos-Villegas et al., 2021). miRNAs mainly act by targeting transcripts of tumor factors or proto-oncogenes (Wang et al., 2008). Therefore, miRNAs can be divided into oncogenic miRNAs (oncomiRs) and tumor suppressor miRNAs (tsmiRs) (Svoronos et al., 2016). OncomiR is usually highly expressed to promote tumor progression and maintain tumor phenotype and is mostly upregulated in cancer, whereas tsmiR is mainly used to regulate cell proliferation and invasion and to promote apoptosis, thereby suppressing tumorigenesis, and is mostly downregulated in cancer (Ali Syeda et al., 2020). Multiple miRNAs have been found to show aberrant expression in CC. For example, miR-92a and miR-494 have been identified as being upregulated in CC patients. Investigators have confirmed using ROC curves that miR-92a-5p expression has high specificity and sensitivity at the optimal threshold in the detection of both low-grade squamous intraepithelial lesions (LSILs) and high-grade squamous intraepithelial lesions (HSILs) (LSILs: 95% sensitivity, 87% specificity; HSILs: 94% sensitivity, 87% specificity) (Azimi et al., 2021). Wang et al. combined qRT-PCR, luciferase assays, and rescue experiments to confirm that miR-92a, which is significantly upregulated in CC tissues, is negatively correlated with its downstream target gene *PIK3RI* (Wang et al., 2021). Liu et al. found a negative relationship between the overall survival of CC patients and miR-92a-3p, and observed that miR-92a-3p is able to promote CC stem cell proliferation, invasion, and cell cycle transition (Liu et al., 2021b). Another study found that miR-494 is positively correlated with the survival time of CC patients (Wen et al., 2022). When miR-494 was used to regulate LETMD1 expression, the proliferation, differentiation, and migration rates of HeLa cell lines increased slowly over 5 days, while the proportion of cancer cells decreased by 5%, the proportion of macrophages increased by 2%, and the proportion of dendritic cells increased by 3% after the expression of LETMD1 (Wen et al., 2022). In an *in vitro* experiment, Yang et al. found that downregulation of miR-494 expression inhibited CC cell proliferation and growth by regulating the expression of target gene *PTEN*, suggesting a potentially significant role of miR-494 in tumorigenesis and the development of CC, and introducing a new perspective for understanding of the molecular mechanisms underlying CC progression (Yang et al., 2015).

In addition, miR-195 and miR-99a have been found to be downregulated in CC patients. Jin et al. showed that miR-195 is negatively correlated with BCDIN3D, as examined by qRT-PCR, and that both are able to inhibit the ki67 protein, a biomarker of CC cell proliferation (Jin et al., 2021). Sun et al. verified that miR-195–5p can substantially lower the expression of PFKFB4 and thus slow down the proliferation of CC cells (Sun and Jin, 2022). Han et al. co-transfected the vectors expressing miR-99a and IGFIR into cells and found that miR-99a can specifically inhibit IGF1R expression, which in turn inhibits CC proliferation and migration (Han, 2021). Similarly, Wang et al. found a negative association between miR-99a-5p and RRAGD, and observed that miR-99a-5p can enable its target gene *RRAGD* to induce apoptosis and inhibit glycolysis in CC cells, representing a potential therapeutic target in CC (Wang et al., 2022b). We summarize the roles of the most recently discovered pro-tumor and anti-tumor miRNAs in CC in Table 3 and Table 4.

**TABLE 3:** Ectopic expression of oncogenic miRNAs in CC.

miRNA	Expression	Types of CC tissues or cell lines studied	Biological function	Mechanism	Reference
miR-1254	Up	30 paired cancerous and normal cervical tissue samples; cells (Ect1/E6E7, HeLa, C33a, SiHa, and CaSki)	Attenuates CC cell invasion and proliferation	Intercalates with CD36 messenger RNA and modulates CD36	Zhang et al. (2022e)
miR-3653	Up	136 patients (CC tissue and adjacent non-cancerous tissue); cells (HaCaT, C33A, SiHa, and HeLa)	Promotes CC progression	Targets Zeb2 or ITGB1	Cui et al. (2022)
miR-34a	Up	Cells (SiHa, HeLa, C33A, and HEK293T)	Promotes CC proliferation, invasion, and migration	Suppresses viral E6 protein and destabilizes overexpressed oncoprotein Cdt2	Singh et al. (2022)
miR-146a-5p	Up	30 patients (CC tissue and adjacent non-cancerous tissue); cells (HEK293T, HeLa, CaSki, SiHa, C33A, End1/E6E7, and HcerEpic)	Promotes CC invasion, EMT, and migration	Negatively regulates WWC2 and activates Hippo-YAP signaling pathway	Wang et al. (2022c)
miR-1323	Up	Cells (HeLa, SiHa, CaSki, C33A, and End1/E6E7)	Promotes CC proliferation, migration, and invasion	Targets PABPN1 to recruit IGF2BP1, thereby further regulating GSK-3β and affecting the Wnt/β-catenin signaling pathway.	Fang et al. (2022)

**TABLE 4:** Ectopic expression of antitumor miRNAs in CC.

miRNA	Expression	Types of CC tissues or cell lines studied	Biological function	Mechanism	Reference
miR-195–5p	Down	TCGA database	Inhibits malignant progression of CC cells	Decreases and restrains PFKFB4; tumor-suppressive effect of miR-195–5p is partially restored by overexpression of PFKFB4	Sun and Jin (2022)
miR-26a-5p	Down	15 patients (CC tissue and para-carcinoma tissue samples); cells (GH329, C33A, HeLa, and SiHa)	Inhibits CC proliferation, migration, and invasion	Negatively correlates with HSDL2 and directly targets HSDL2	Li et al. (2022a)
miR-106b-5p	Down	80 patients (CC tissue and adjacent tissue); cells (SiHa, C-33A, ME-180, MS-751, HCC-94, HeLa, and HEK-293 T)	Suppresses CC cell proliferation, migration, and invasion	Negatively regulates FGF and inhibits CC growth and metastasis by downregulating FGF4 expression	Lei et al. (2022)
miR-423–3p	Down	Cells (HeLa, CaSki, SiHa, and Ect1/E6E7)	Suppresses CC cell progression and tumor growth	Regulates macrophage M2 polarization by targeting CDK4 mRNA and inhibits phosphorylation of STAT3 via CDK4 to silence IL-6 expression	Yan et al. (2022)
miR-218–5p	Down	TCGA, GEO, and HPA databases	Inhibits CC cell migration, invasion, and proliferation	Negatively regulates the target gene RUNX2 and participates in positive modulation of CC cell proliferation	Cruz-De la Rosa et al. (2022)
miR-331–3p	Down	98 patients (CC tissue and adjacent tissue); cells (CaSki, C33A, HeLa, SiHa, and HEK293T)	Inhibits CC cell growth and malignant progression	Inhibits the expression of DNMT3A by binding to DNMT3A mRNA; DNMT3A promotes LIMS2 methylation and reduces expression of LIMS2	Yang et al. (2022c)

## 4 The mechanisms underlying lncRNA–miRNA interactions in CC

lncRNAs and miRNAs interact with one other in the development of CC via the following mechanisms.

lncRNA competes with miRNAs to complementarily bind target mRNA with the help of the miRNA response element (MRE) and enables release of the negative regulation of target genes by miRNA to enhance its stability. Li et al. found that miR-148a-3p negatively regulates c-Met by binding to the 3′ UTR of c-Met, while small nuclear host gene 4 (SNHG4) exerts a protective effect on the target mRNA by competitively binding miR-148a-3p to the 3′ UTR of c-Met (Li et al., 2019b).

miRNAs can negatively regulate lncRNAs in a manner similar to the mechanism of mRNAs, specifically by using the principle of base complementation to recognize lncRNAs and reduce lncRNA stability with the cooperation of RISC. Zhang et al. (2013) found that miR-21 interacts with growth arrest-specific transcript 5 (GAS5) and regulates it in a manner similar to miRNA-mediated silencing of target mRNAs, which explains the ability of miR-21 to target both tumor suppressor genes and the lncRNA GAS5.

lncRNAs take the form of potential precursor miRNAs (pre-miRNAs). lncRNAs are first cleaved by class 2 ribonuclease III (Drosha enzyme) in the nucleus to form precursors of mRNAs. Some genes produce lncRNAs and miRNAs at the same time, but these miRNAs are immature and need to be processed by endoribonuclease (Dicer) in the cytoplasm before eventually transforming into mature miRNAs. Keniry et al. (2012) suggested that lncRNA H19 produces miR-675 during the dynamic regulation of RNA-binding protein (HuR) related to stress response.

lncRNAs act as sponges for miRNA to inhibit the binding of miRNAs to mRNAs and to facilitate the degradation of lncRNAs. Li et al. found that the expression of lncRNA DSCAM-AS1 in cervical cancer specimens was negatively correlated with miR-338–3p expression, while upregulation of DSCAM-AS1 decreased miR-338–3p expression in SiHa cells, which, in turn, promoted the growth, cycle, and invasion of cervical cancer cells, suggesting that lncRNA DSCAM-AS1 could sponge adsorption of miR-338–3p and thus could play a pro-cancer role in cervical cancer (Li et al., 2022b).

miRNAs can target DNA methyltransferase (DNMT) to regulate lncRNA expression levels. Chen et al. found that DNA methyl-transferase 1 (DNMT1) is a target gene of miR-148a-3p and that it can regulate level of expression of transcription factor-1 (UTF1) in cervical cancer cells, while promoter hypermethylation is necessary for the initiation of UTF1 expression (Chen et al., 2021).

**FIGURE 3:**
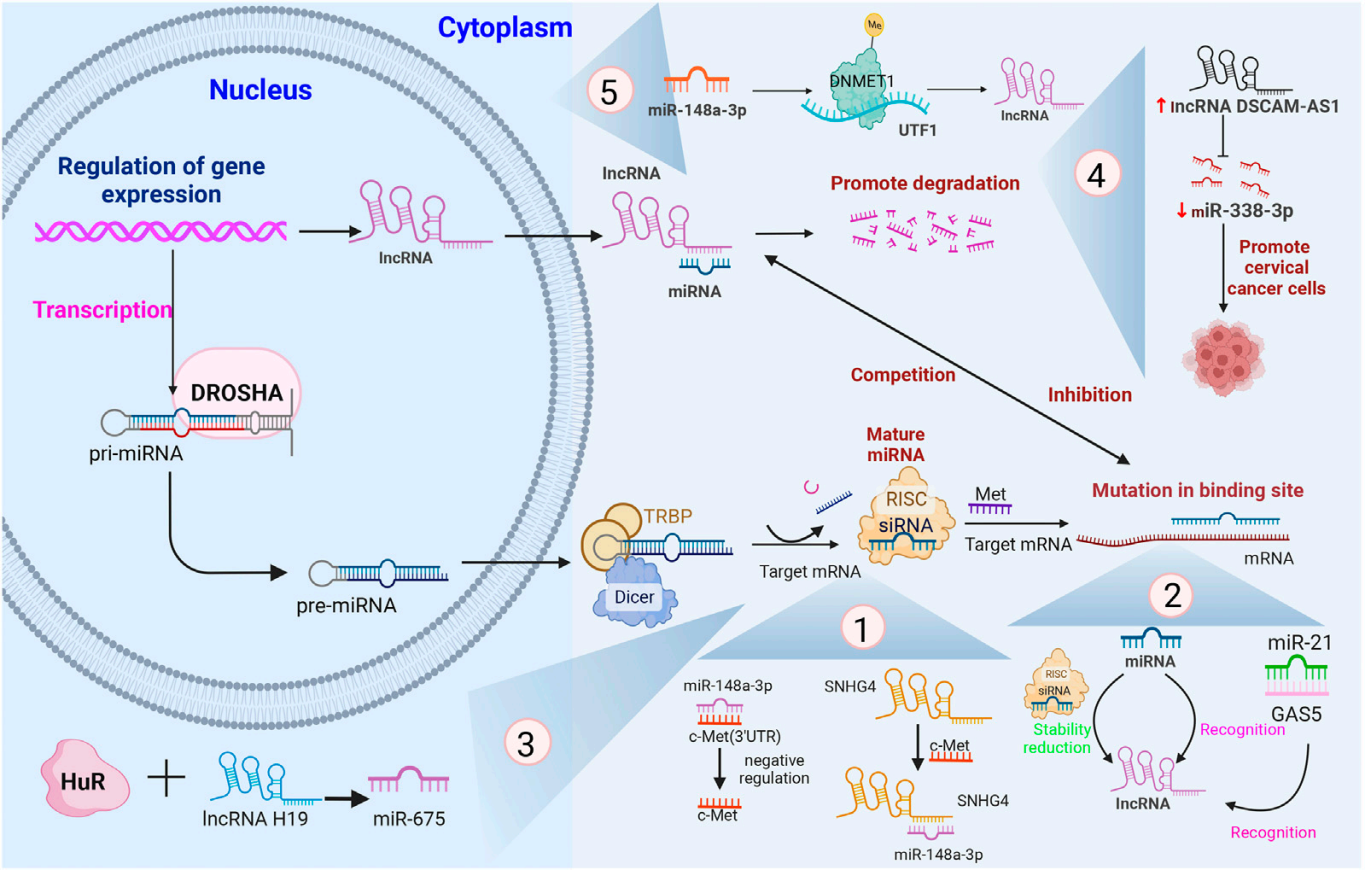
The five different mechanisms involving lncRNA–miRNA interactions in CC and their associated processes, with several specific examples that have been shown to be involved in the different mechanisms. Note: pri-miRNA, primary miRNA; pre-miRNA, precursor miRNA; RISC, RNA-induced silencing complex; 3′-UTR, 3′-untranslated regions; TRBP, TAR RNA-binding protein; GAS5, growth arrest-specific transcript 5; DNMT1, DNA methyltransferase 1; UTF1, undifferentiated embryonic cell transcription factor-1; SNHG4, small nuclear host gene 4; DSCAM-AS1, DSCAM antisense RNA 1.

The five distinct mechanisms underlying lncRNA–miRNA interactions in CC are illustrated in Figure 3.

## 5 Important roles of lncRNA- and miRNA-related signaling pathways in CC

Although combination therapies are used with CC patients, their overall therapeutic efficacy does not meet expectations. lncRNAs and miRNAs play pro-tumor or anti-tumor roles by activating or inhibiting certain pathways. Therefore, interfering with lncRNA- and miRNA-related signaling pathways may provide a direction for the development of further treatment for CC.

### 5.1 Phosphatidylinositol 3-kinase/protein kinase B (PI3K/Akt) signaling pathway

#### 5.1.1 Mechanism underlying the PI3K/Akt pathway

PI3K activation originates from various stimulating factors such as VEGF, fibroblast growth factor (FGF), and insulin, which activate receptor protein tyrosine kinase (RPTK), causing auto-phosphorylation (Laddha and Kulkarni, 2019; Acosta-Martinez and Cabail, 2022). The catalytic subunit p110 in the PI3K structure catalyzes the formation of phosphatidylinositol biphosphate (PIP2) on the plasma membrane to produce the second messenger phosphatidylinositol trisphosphate (PIP3) (Acosta-Martinez and Cabail, 2022). PIP3 transports Akt to the plasma membrane to form a complex, thereby further activating Akt for regulation of downstream target proteins in the form of an activated or suppressed effect and thus regulating cancer cell proliferation and apoptosis (Acosta-Martinez and Cabail, 2022).

#### 5.1.2 lncRNA–miRNA interaction regulates CC through the PI3K/Akt signaling pathway

The PI3K/Akt pathway can become involved in apoptosis, proliferation, invasion, and migration. In recent years, numerous studies have confirmed that abnormal activation of the PI3K/Akt pathway is a significant mechanism in tumor pathogenesis, including that of cervical cancer. Li et al. found that KCNQ1OT1 upregulation increases SiHa viability but inhibits its apoptosis; miR-1270 mimics lead to low viability and high apoptosis in SiHa cells, while LOXL2 overexpression promotes SiHa cell viability and reduces apoptosis (Jiang et al., 2022). LOXL2, the target of miR-1270, positively interacts with KCNQ1OT1 but has a negative interaction with miR-1270 (Jiang et al., 2022). Additionally, lncRNA KCNQ1OT1 activates the PI3k/Akt pathway by sponging miR-1270 to alter the expression of the target gene LOXL2, thereby initiating apoptosis and promoting the development of CC (Jiang et al., 2022). Finally, it has previously been reported that high expression of LINC00673 could act as a “miRNA sponge” in its effect on miR-126–5p, thereby enhancing PTEN protein expression and activating the PI3K/Akt signaling pathway to promote CC cell proliferation (Shi et al., 2020).

### 5.2 Wnt/β-catenin signaling pathway

#### 5.2.1 Mechanism of the Wnt/β-catenin pathway

Wnt ligands are secreted glycoproteins that recognize and bind to the corresponding membrane protein receptor, which leads to β-catenin accumulation, thereby avoiding phosphorylation by glycogen synthase kinase-3β (GSK-3β) (Hadi et al., 2020; Hayat et al., 2022). The non-phosphorylated β-catenin can avoid degradation and ubiquitination by the intracytoplasmic damage complex and can thus gradually accumulate in the cytoplasm and translocate to the nucleus (Nalli et al., 2022). Subsequently, non-phosphorylated β-catenin in the nucleus binds to the transcription factor T-cell factor/lymph enhancer factor (TCF/LEF) to activate the downstream cellular myelocytomatosis viral oncogene (c-Myc), leading to cancer cell proliferation and differentiation (Zhang and Wang, 2020; Nalli et al., 2022). This indicates that the β-catenin-TCF/LEF complex is key in activation of the Wnt/β-catenin pathway.

#### 5.2.2 lncRNA–miRNA interaction regulates CC through the Wnt/β-catenin signaling pathway

Family with sequence similarity 201 member A (FAM201A) enhances cell viability, migration, and invasion in CC *in vivo*. Wang et al. found that high expression of FAM201A can upregulate FLOT1 expression through sponging of miR-1271–5p, which in turn activates the Wnt/β-catenin pathway to promote CC progression and metastasis (Wang et al., 2022d). Furthermore, Niu et al. demonstrated that AXIN2, a target gene of miR-205–5p, negatively regulates activation of the Wnt/β-catenin pathway (Chen et al., 2021; Niu et al., 2021). DKK1 and β-catenin are markers of the Wnt/β-catenin pathway. Low expression of HNRN-PU-AS1 and high expression of miR-205–5p can promote β-catenin expression and inhibit DDK1 expression, thereby activating the Wnt/β-catenin pathway. Moreover, high expression of AXIN2 inhibits activation of the Wnt/β-catenin pathway, thereby suppressing cell proliferation and promoting apoptosis in CC (Niu et al., 2021).

### 5.3 Mitogen-activated protein kinase/extracellular signal-regulated kinase (MEK/ERK) signaling pathway

#### 5.3.1 Mechanism of the MEK/ERK signaling pathway

MEK1/2 and ERK1/2 (referred to as MEK and ERK) are two highly conserved and functionally similar heterodimers. MEK1/2 has very narrow substrate specificity and catalyzes the phosphorylation of tyrosine and threonine residues on the TEY motif of ERK1/2 (Barbosa et al., 2021). ERK1/2 is regarded as a typical mitogen-activated protein kinase (MAPK) involved in signal transduction and transcriptional regulation, which can activate nuclear and cytosolic targets and participate in negative feedback loops (Wu et al., 2020). ERK1/2 has broad substrate specificity and is the only known downstream target of MEK1/2 (Barbosa et al., 2021). The interaction mechanism of ERK kinases in mediating the downstream targets of MEK activation contributes to the functional operation of the MEK/ERK pathway (Degirmenci et al., 2020).

#### 5.3.2 lncRNA–miRNA interaction regulates CC through the MEK/ERK signaling pathway

The MEK/ERK signaling pathway is involved in regulating the physiological and pathophysiological processes of CC; specifically, inhibition of MEK induces the inactivation of ERK1/2, thereby reducing tumor cell proliferation and promoting apoptosis. Guo et al. detected significantly elevated expression of SNHG20 in CC via qRT-PCR assay and found that overexpression of miR-140–5p, a downstream target of SNHG20, inhibits MEK/ERK signaling, which, in turn, suppresses the proliferation and invasive ability of CC cells (Guo et al., 2018). In an *in vivo* experiment, Zhang et al. observed that LOXL1-AS1 downregulation inhibits tumor growth, metastasis, and proliferation of CC cells (Zhang et al., 2022f). Further investigation of the mechanism revealed that LOXL1-AS1 can regulate expression of the target gene ENC1 by sponging miR-423–5p. In addition, knockdown of ENC1 was found to inhibit activation of the ERK/MEK pathway based on measurements of the corresponding protein levels. Consequently, the authors concluded that the LOXL1-AS1/miR-423–5p/ENC1 axis accelerates CC development through the MEK/ERK signaling pathway, introduction a new direction of study in terms of the molecular basis of CC (Zhang et al., 2022f).

## 6 Summary

In summary, ncRNAs can exert an effect on CC development through a variety of molecular mechanisms. Significant progress has been made in the study of lncRNAs and miRNAs, mainly focusing on regulation of cell cycle, EMT, and angiogenesis by lncRNAs and miRNAs, among other aspects. In the future, we should continue to explore the mechanisms of lncRNAs and miRNAs in the development of CC, focusing on the results of current research. In particular, we need to clarify the effects of lncRNA–miRNA interactions on the mechanisms underlying CC development and establish further evidence relating to their potential role in early diagnosis of CC. However, the role of lncRNAs and miRNAs in signaling pathways related to CC also needs to be further explored. Numerous studies have shown that lncRNAs and miRNAs are closely related to tumor-related signaling pathways and can be used as target genes to induce the activation and transduction of certain signaling pathways. For example, PI3K/Akt, Wnt/β-catenin, and MEK/ERK signaling pathways can play a role in the processes of CC cell growth, proliferation, and metastasis. The normal operation of signaling pathways relies on a sophisticated network and the functioning of each tiny link. If there is a disruption in one of the links, a change in signaling pathways can occur, which will result in the alteration of oncogenes and anti-tumor genes, promoting cancer infiltration and metastasis. Risk factors, together with the regulation of intercellular signaling pathways, affect tumor growth by enabling evasion of immune system surveillance and clearance, inducing EMT, angiogenesis, and regulation of the cell cycle. Currently, due to the inadequacy of screening and long period of latency associated with CC, it is necessary to strengthen research on lncRNAs and miRNAs and the signaling pathways associated with them in order to achieve major improvements in the diagnosis and treatment of CC.
